# Nontraumatic Laryngeal Fractures: Report of Two Cases and Review of the Literature

**DOI:** 10.1155/2017/2153521

**Published:** 2017-04-09

**Authors:** Alfredo Santamaría, Ricardo Alarcón, Ilson Sepúlveda, Felipe Fredes

**Affiliations:** ^1^ENT-Head and Neck Surgery Service, General Hospital of Concepcion, University of Concepcion School of Medicine, Concepcion, Chile; ^2^Radiology Department, ENT-Head and Neck Surgery Service, General Hospital of Concepcion, Concepcion, Chile

## Abstract

Laryngeal fractures occur mainly in the context of cervical trauma, hanging, or strangulation. Nontraumatic laryngeal fractures are rare and there are few reports in the literature. We present two cases of nontraumatic laryngeal fractures evaluated in our service.

## 1. Introduction

Laryngeal fractures occur mainly from direct trauma, suicide by hanging, or strangulation [1]. Nontraumatic laryngeal fractures are rare, with 4 cases reported in the literature [2–5]. We present two cases of nontraumatic laryngeal fractures, the first case after a sneeze attack and the second case after swallowing.

## 2. Case Reports

### 2.1. Case 1

A 36-year-old male patient presented to the emergency department with severe odynophagia and dysphonia. The patient had no significant prior medical or surgical history, except for a traffic accident 12 years ago resulting in complicated abdominal trauma with no cervical lesions. He reported that the symptoms started two hours ago and had an abrupt onset after a contained sneeze in a work meeting. He reports that after the sneeze he felt a crack in the neck and after that the symptomatology was installed.

**Figure 1:**
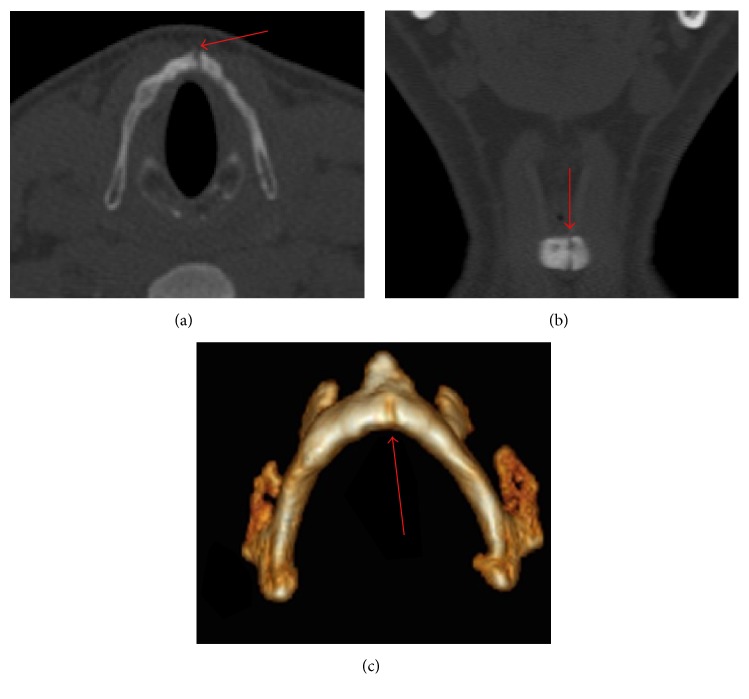
Computed tomography, bone window. Left, complete, and nondisplaced parasagittal fracture that compromises thyroid cartilage (red arrows). (a) Axial view; (b) coronal view; (c) 3D bone reconstruction.

It was evaluated in the otolaryngology department, on physical examination, that the oropharynx and neck examination revealed no tonsillar erythema or edema but diffuse tenderness over the thyroid cartilage without subcutaneous emphysema. A nasopharyngolaryngoscopy was performed, in which a left vocal fold hematoma was evidenced with normal vocal fold movement. The study was completed with a computed tomography, observing a left, complete, nondisplaced parasagittal fracture that compromises thyroid cartilage (Figure 1).

It was managed with oral corticosteroids for 7 days and vocal rest for 15 days, achieving complete resolution of symptoms in 21 days. At the 5-year follow-up, the patient has not had another episode.

### 2.2. Case 2

A 32-year-old male patient with no significant prior medical or surgical history presented to the otolaryngology service with odynophagia, dysphagia, and dysphonia. The symptoms started six days ago and had an abrupt onset after swallowing and bending over during dinner. He reports that after that he felt a crack in the neck and then the symptomatology was gradually installed.

**Figure 2:**
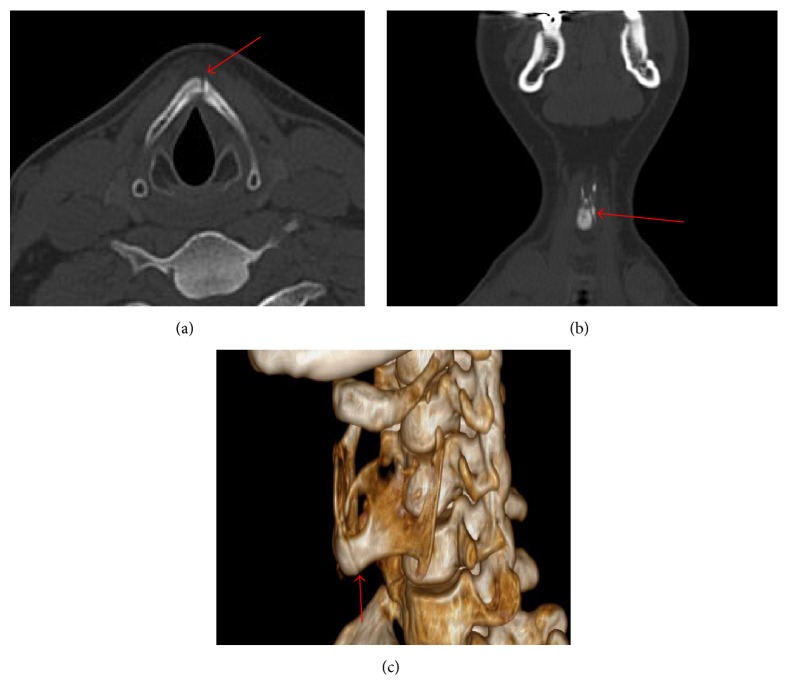
Computed tomography, bone window. Left, complete, and nondisplaced parasagittal fracture that compromises thyroid cartilage (red arrows). (a) Axial view; (b) coronal view; (c) 3D bone reconstruction.

On physical examination the patient had pain over the thyroid cartilage without subcutaneous emphysema. A nasopharyngolaryngoscopy was performed, in which supraglottic edema with normal vocal fold movement was evidenced. The study was completed with a computed tomography that showed an anterior, left parasagittal, complete nondisplaced thyroid cartilage fracture (Figure 2).

It was managed with nonsteroidal anti-inflammatory drugs and vocal rest for 5 days, achieving complete resolution of symptoms in 10 days. The patient has not had another episode at the 3-month follow-up.

## 3. Discussion

Nontraumatic laryngeal fractures are a rare pathology, with only 4 cases published in the literature, all of which are isolated case reports [2–5]. Our series is the first to bring two cases treated in the same center.

All the published cases correspond to men without a prior morbid or surgical history with a mean age of 40 years (range 29–47 years) [5]. Our two cases were men of 36 and 32 years, respectively.

Its etiology is still unknown. A congenital anomaly of the laryngeal cartilage is proposed, associated with an alteration in the mineralization and ossification, producing sites of focal weakness that predisposes them to develop fractures [4]. However, due to the small number of cases, this has not been demonstrated.

Clinically, the patients presented with the symptomatic triad of dysphonia, dysphagia, and odynophagia initiated after a precipitating event. The precipitating events described in the literature are sneezing attacks in two cases [2, 3] and after coughing in the other two cases [4, 5]. All patients reported having experienced a crack after the precipitating event, followed by the clinical manifestations described. The physical examination highlights the pain over the thyroid cartilage [2–5] and the presence of subcutaneous emphysema [2, 4]. In our cases the symptomatic triad was presented in one case and in the other only odynophagia and dysphonia; both reported having felt a crack at the beginning of the symptomatology. As for the precipitating event, in one case it was a sneeze and the other was after swallowing while bending down with the neck in flexion, this being the only case described triggered by this mechanism.

It is fundamental to perform a nasopharyngolaryngoscopy and a neck computed tomography to confirm the diagnosis [3–5]. These exams were performed in all cases, except the first one described in the literature in 1950 [2], where these exams were not available. This patient was evaluated by direct laryngoscopy and chest X-ray [2]. The most frequent endoscopic findings were edema and unilateral vocal fold hematoma with preserved vocal fold movement [5]. In the computed tomography, subcutaneous emphysema was evidenced, nondisplaced, or mildly displaced laryngeal fractures, and in one case a phlegmon was also observed; in this case the patient was coursing a high respiratory infection at the time of fracture [5].  Table 1 summarizes the findings of all cases published in the literature plus our two cases.

The treatment basically consists of observation, anti-inflammatory, and vocal rest, achieving complete resolution of the symptoms between 14 and 21 days. A single case received antibiotics because it was associated with a cervical phlegmon. As all fractures were not displaced or mildly displaced, none required reduction with titanium plates [5].

## 4. Conclusion

Nontraumatic laryngeal fracture is a rare condition, affecting men between the third and fifth decade of life, which should be suspected in patients with the symptomatic triad of odynophagia, dysphagia, and dysphonia abruptly initiated after a precipitating event such as coughing or sneezing. Clinical suspicion, endoscopy, and imaging are fundamental for diagnosis.

## Figures and Tables

**Table 1:** Symptoms, signs, and endoscopic and imaging findings among patients with nontraumatic laryngeal fracture.

	Case 1 [2]	Case 2 [3]	Case 3 [4]	Case 4 [5]	Case 5	Case 6
Age/sex	44/male	29/male	41/male	47/male	36/male	32/male

Precipitating event	Sneeze	Sneeze	Cough	Cough	Sneeze	Swallowing and bending over

Symptoms/signs	Odynophagia, dysphagia, dysphonia. Pain and crepitus over the thyroid cartilage	Odynophagia and dysphonia	Odynophagia, dysphagia, dysphonia. Crepitus over the thyroid cartilage	Odynophagia, dysphagia, dysphonia. Pain over the thyroid cartilage	Odynophagia and dysphonia. Pain over the thyroid cartilage	Odynophagia, dysphagia, dysphonia. Pain over the thyroid cartilage

Laryngoscopy	Supraglottic edema, normal vocal fold movement	Right true vocal fold edema, mucosal hematoma, normal vocal fold movement	Left true vocal fold hematoma, left ventricular edema, normal vocal fold movement	Edema of right aryepiglottic fold and both arytenoids, normal glottis, normal vocal fold movement	Left vocal fold hematoma, normal vocal fold movement	Supraglottic edema, normal vocal fold movement

Computed tomography (CT)	Not available	Nondisplaced anterior fracture, subcutaneous air	Mildly displaced anterior fracture, subcutaneous air	Mildly displaced anterior fracture, subcutaneous air. Phlegmon formation* *	Left parasagittal, complete nondisplaced thyroid cartilage fracture	Left parasagittal, complete nondisplaced thyroid cartilage fracture
